# Employment status and barriers to workforce participation among individuals with spinal cord injury: results from the Turkish InSCI Community Survey

**DOI:** 10.1038/s41393-026-01194-1

**Published:** 2026-03-19

**Authors:** Belgin Erhan, Bilinc Dogruoz Karatekin, Kevser Gumussu, Basak Bilir Kaya, Hulya Sirzai, Yasemin Yumusakhuylu, The Spinal Cord Paralytics Association of Turkey, Semra Çetinkaya

**Affiliations:** 1 Department of Physical Medicine and Rehabilitation, Istanbul Medeniyet University Faculty of Medicine, Istanbul, Turkey; 2 Physical Medicine and Rehabilitation, Goztepe Prof Dr Suleyman Yalcin City Hospital, Istanbul, Turkey; 3 Department of Physical Medicine and Rehabilitation, Istanbul Gaziosmanpasa Training and Research Hospital, Istanbul, Turkey; 4 Erenköy Physical Medicine and Rehabilitation Hospital, Istanbul, Turkey; 5 Romatem Physical Medicine and Rehabilitation Hospital, Istanbul, Turkey; 6 The Spinal Cord Paralytics Association of Turkey, Istanbul, Turkey

**Keywords:** Population screening, Spinal cord diseases, Rehabilitation

## Abstract

**Study design:**

Cross-sectional survey.

**Objectives:**

To evaluate the employment status, work environment perceptions, and reasons for unemployment among individuals with SCI in Turkey, and to identify factors associated with workforce participation.

**Setting:**

Community.

**Methods:**

Data were collected from 357 individuals with SCI using the standardized International Spinal Cord Injury (InSCI) Community Survey. Data on sociodemographic and SCI-related characteristics, employment status, vocational rehabilitation (VR) participation, income satisfaction, and perceived barriers to employment were collected. Group differences between employed and unemployed participants were analyzed.

**Results:**

The mean age of participants was 44.97 ± 10.92 years, and 71.7% were male. Only 12.9% were currently engaged in paid employment, while 59.5% of unemployed participants expressed a willingness to work. Employment was significantly associated with higher education (p < 0.001), more education received after injury (p < 0.001), longer time since injury (p < 0.001), and participation in VR (p = 0.003). The most common reasons for not working were health condition or disability (67.7%), inability to find a suitable job (20.7%), insufficient transportation services (14.8%), and lack of knowledge about job-seeking strategies (14.5%). Fear of losing disability benefits was reported by 3.6% of participants. Among those employed, 52.3% reported satisfaction with their salary, and 78.3% felt that their contributions were adequately recognized.

**Conclusions:**

Employment rates among individuals with SCI in Turkey remain critically low, despite many expressing a desire to work. Health-related limitations, environmental barriers, and systemic issues such as transportation and lack of VR services are key factors hindering workforce participation.

## Introduction

Spinal cord injury (SCI) profoundly disrupts individuals’ physical function, social roles, and economic participation, making employment a particularly critical outcome for quality of life and psychological well-being [1]. Studies consistently show that individuals with SCI face marked challenges in returning to or maintaining work, even when motivated and capable [2–4].

Early reviews highlight that employment rates post-SCI vary widely, typically ranging between 11 and 74%, depending on factors such as injury severity, age at onset, and time since injury [4]. Recovery trajectories improve gradually, with return-to-work (RTW) more likely among those with younger age at injury and higher functional independence [3, 4]. A comprehensive meta-review reported an average post-SCI employment rate of approximately 35%, substantially lower than the general population [1].

Employment after SCI is influenced by multiple individual and environmental factors. Vocational rehabilitation (VR) is a key intervention aimed at addressing these challenges. VR is considered an important component of RTW after SCI, aiming to facilitate work participation through individualized support and workplace-related interventions. VR systems and practices vary widely across countries, and differences in timing, funding, and accessibility can influence outcomes [5, 6]. Understanding these factors is essential to developing effective interventions tailored to local contexts.

In Turkey, prior research has identified employment rates of around 21% among individuals with SCI, with predictors of employment mirroring international patterns [3].

Building on this body of literature, our study sought to quantify the current employment rate among a Turkish cohort of individuals with SCI, examine individual-level and contextual determinants (including education, time since injury, VR participation, injury type, and gender), and elucidate the perceived barriers to employment. By comparing our findings with both international benchmarks and regional research, we aim to contribute to a more contextually nuanced understanding of employment dynamics after SCI and to inform interventions tailored to our setting.

## Methods

### Study design and data source

This study was designed as a cross-sectional, observational analysis utilizing data from the Turkish arm of the International Spinal Cord Injury (InSCI) Community Survey. The InSCI survey is a standardized, international initiative developed under the framework of the WHO’s Global Disability Action Plan, aiming to assess the lived experiences, health conditions, and societal participation of individuals with SCI across diverse sociocultural settings. The Turkish dataset used in this study adheres to the core methodology and standardized structure implemented across all participating countries, allowing for international comparability.

Ethical approval for this study was obtained from the Clinical Research Ethics Committee of S.B. Istanbul Medeniyet University Göztepe Research and Training Hospital on December 22, 2021 (Approval No: 2021/0681). Written informed consent was secured from all participants prior to enrollment, in compliance with the Declaration of Helsinki.

### Participants

**Fig. 1:**
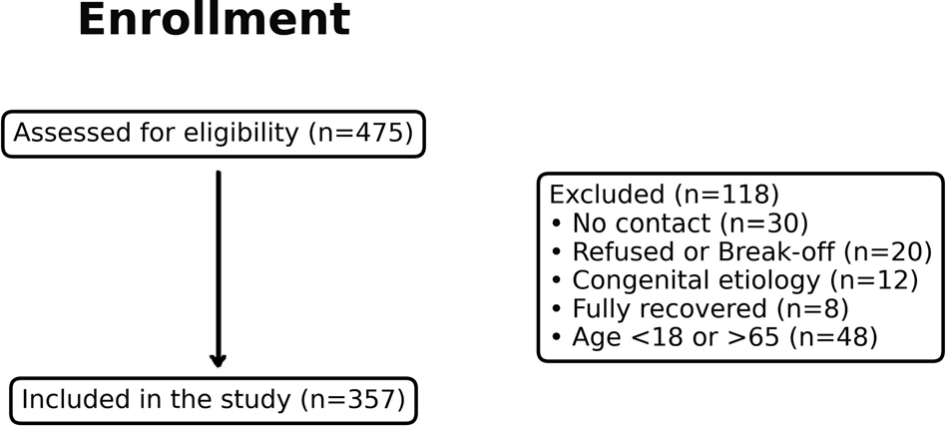
Participant flow of the study cohort. Flow diagram showing the screening, eligibility assessment, exclusion of participants outside the working-age range (18–65 years), and final inclusion of 357 community-dwelling individuals with spinal cord injury in the analysis.

A total of 475 community-dwelling individuals with SCI were screened for eligibility, of whom 405 met the general inclusion criteria. As the study focused on the working-age population (18–65 years), participants outside this age range were excluded, resulting in a final sample of 357 individuals included in the analysis (Fig. 1).

Inclusion criteria were as follows:

- History of traumatic or non-traumatic SCI,
- Residence in the community (non-institutionalized) in Turkey,
- Ability to provide informed consent and to complete the survey either independently or with assistance.

Exclusion criteria included congenital causes of SCI, neurodegenerative conditions, and cognitive impairment preventing comprehension of the survey.

Participants were recruited from five major centers in Istanbul, including one university hospital (public), two specialized rehabilitation centers (one public and one private), and one national disability association (The Spinal Cord Paralytics Association of Turkey). Although all institutions are based in Istanbul, they serve individuals from across Turkey. Recruitment was monitored to ensure proportional representation from all geographical regions of the country, thereby enhancing the generalizability of the findings.

### Data collection

Data were collected using the official Turkish version of the InSCI questionnaire. The survey was administered either as a self-reported paper or electronic questionnaire, or through structured face-to-face interviews when necessary. Standardized InSCI procedures were strictly followed to maintain consistency and reliability across sites.

For this study, data collection focused on the following domains:

1. Sociodemographic characteristics: age, gender, marital status, education level, income, and disability pension status.
2. SCI-related characteristics: injury type (traumatic/non-traumatic), neurological level, completeness, cause, and time since injury.
3. Employment history and status: current employment, previous employment before SCI, VR participation, income satisfaction, and perceived barriers to employment.
4. Work environment perceptions: availability of assistive devices, recognition at work, salary adequacy, and willingness to work in paid employment.
5. Barriers to employment: assessed through a multiple-response module identifying reasons for unemployment (e.g., health condition, lack of suitable jobs, transportation barriers).

Definition of paid employment: “Currently in paid employment” was defined as self-reported engagement in paid work at the time of survey administration. Paid work included any remunerated activity (wage/salary employment and self-employment), irrespective of full-time or part-time status. Participants not reporting current paid work were classified as not currently in paid employment.

Income satisfaction: Income satisfaction was recorded on a 0–10 scale, with higher scores indicating greater satisfaction with income.

Reasons for not working: Reasons for not working were assessed using a standardized multiple-response item list (participants could select all applicable reasons), including “fear of losing disability benefits” as one selectable option.

### Statistical analysis

All statistical analyses were performed using IBM SPSS Statistics version 25.0 (IBM Corp., Armonk, NY, USA). Descriptive statistics, including means, standard deviations, frequencies, and percentages, were calculated to summarize sociodemographic variables, SCI characteristics, employment rates, and reported barriers to work.

Comparisons between employed and unemployed participants were conducted using independent samples t-tests for continuous variables (e.g., age, years of education, SCI duration, income satisfaction) and chi-square tests for categorical variables (e.g., gender, type of injury, VR participation).

A two-tailed p-value < 0.05 was considered statistically significant. Missing data were handled using pairwise deletion to maximize available information for each analysis.

## Results

A total of 357 individuals with SCI participated in the study. The mean age of the participants was 44.97 ± 10.92 years (range: 23–65 years), and 71.7% were male. Regarding marital status, 47.6% were married, while 41.2% were single. In terms of education, 33.1% had completed primary school, and 11.2% were university graduates. The average years of education were 9.45 ± 4.84, with 2.44 ± 4.26 years of education occurring after the onset of SCI. More than half of the participants (57.1%) reported working before their SCI, whereas only 12.9% were currently employed in paid work. Additionally, 54.6% were receiving a disability pension, and 72.6% reported an income above the minimum wage (Table 1).

**Table 1:** Sociodemographic characteristics of the participants (n = 357).

Characteristic	n (%) / mean ± SD (min-max)
**Age**	44.97 ± 10.92 (23–65)
**Gender**	
Male	256 (71.7%)
Female	101 (28.3%)
**Relationship**	
Single	147 (41.2%)
Married	170 (47.6%)
Co-living	15 (4.2%)
Separated	19 (5.3%)
Widowed	6 (1.7%)
**Education**	
Never Attended School	11 (3.1%)
Primary School	118 (33.1%)
Middle School	48 (13.5%)
High School	90 (25.3%)
2-Year Faculty	28 (7.9%)
Left University	10 (2.8%)
University Graduate	40 (11.2%)
Other	11 (3.1%)
**Years of Education**	9.45 ± 4.84 (0–19)
**Years of Education After SCI**	2.44 ± 4.26 (0–16)
**Working Before SCI**	201 (57.1%)
**Currently Working**	46 (12.9%)
**Receiving Disability Pension**	219 (54.6%)
**Income**	
Below Minimum Wage	77 (27.4%)
Above Minimum Wage	204 (72.6%)

*SCI* spinal cord injury

The mean duration of SCI among participants was 27.92 ± 11.35 years (range: 2–59 years). The majority had paraplegia (78.2%), while 20.3% had tetraplegia and 1.4% were fully recovered. Regarding the degree of injury, 51.1% had complete injuries and 48.9% had incomplete injuries. The majority of cases were traumatic (90.3%), with the most common causes being traffic accidents (33.9%), falls greater than 1 meter (18.7%), and work-related accidents (18.7%). The mean visual analogue scale (VAS) score for pain was 4.90 ± 2.52 (Table 2).

**Table 2:** SCI-Related Characteristics (n = 357).

Characteristic	n (%) / mean ± SD (min-max)
**Duration of SCI**	27.92 ± 11.35 (2–59)
**SCI type**	
Paraplegia	277 (78.2%)
Tetraplegia	72 (20.3%)
Fully Recovered	5 (1.4%)
**SCI degree**	
Complete Injury	176 (51.1%)
Incomplete Injury	169 (48.9%)
**Traumatic SCI**	290 (90.3%)
-Sports Injury	3 (0.9%)
-Leisure Activity	10 (3.1%)
-Work Accident	60 (18.7%)
-Traffic Accident	109 (33.9%)
-Violence	26 (8.1%)
-Fall <1 m	5 (1.5%)
-Fall >1 m	60 (18.7%)
-Medical Procedure/Surgery	17 (5.3%)
**Non-Traumatic SCI**	31 (9.7%)
-Degenerative	6 (1.9%)
-Tumor	7 (2.2%)
-Vascular Disease	10 (3.1%)
-Infection	8 (2.5%)
**VAS**	4.90 ± 2.52 (0–10)

*SCI* spinal cord injury, *VAS* visual analogue scale

### Group differences between employed and non-employed participants

Participants who were currently employed had significantly longer SCI duration (21.04 ± 10.33 years vs. 14.32 ± 11.42 years, p < 0.001) and had significantly more years of education (13.13 ± 3.70 vs. 8.26 ± 4.17, p < 0.001). Education received after the SCI was also higher among those currently working (3.60 ± 4.89 vs. 1.01 ± 2.84, p < 0.001).

There was a significant difference in gender distribution, with a higher proportion of females being employed compared to males (p = 0.001). Additionally, individuals with a disability pension were less likely to be employed (p < 0.001), and participation in VR was more common among those currently working (p = 0.003). However, there were no significant differences in SCI type (p = 0.096) or degree of injury (p = 0.967) between the groups (Table 3).

**Table 3:** Group Differences in Sociodemographic and Clinical Variables by Employment Status.

Variable	Currently in paid employment n (%) / Mean ± SD	Not in paid employment n (%) / Mean ± SD	p-value
**Age (years)**	41.58 ± 9.33	42.35 ± 12.44	0.689
**Gender**			**0.001**
Male	23 (50.0)	231 (74.8)	
Female	23 (50.0)	78 (25.2)	
**Education (years)**	13.13 ± 3.70	8.26 ± 4.17	**<0.001**
**Education after SCI (years)**	3.60 ± 4.89	1.01 ± 2.84	**<0.001**
**Income Satisfaction**	4.41 ± 2.56	3.42 ± 2.52	**0.014**
**Income**			0.053
Below Minimum Wage	11 (45.8)	66 (25.7)	
Above Minimum Wage	13 (54.2)	191 (74.3)	
**SCI Duration (years)**	21.04 ± 10.33	14.32 ± 11.42	**<0.001**
**SCI type**			0.096
Paraplegia	32 (69.6)	245 (79.8)	
Tetraplegia	12 (26.1)	59 (19.2)	
Fully Recovered	2 (4.3)	3 (1.0)	
**SCI degree**			0.967
Complete	23 (50.0)	153 (50.3)	
Incomplete	22 (47.8)	146 (48.0)	
Fully Recovered	1 (2.2)	5 (1.6)	
**Vocational Rehabilitation**			**0.003**
Yes	13 (28.3)	36 (11.8)	
No	33 (71.7)	270 (88.2)	
**Disability Pension**			**<0.001**
Yes	14 (30.4)	192 (62.1)	
No	32 (69.6)	117 (37.9)	

*SCI* spinal cord injury

### Perceptions of work environment and satisfaction

**Fig. 2:**
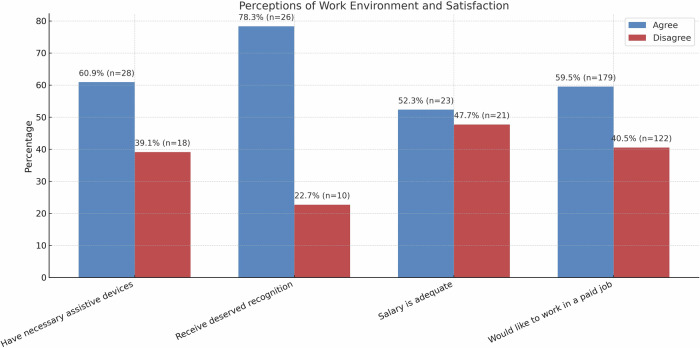
Work environment perceptions and employment-related preferences among individuals with spinal cord injury. The figure shows the proportions of participants reporting availability of necessary assistive devices, perceived recognition at work, satisfaction with salary adequacy, and willingness to work in a paid job.

Among participants, 60.9% reported having necessary assistive devices, while 39.1% indicated that their needs were only partially or not at all met. In terms of job satisfaction, 78.3% agreed that they received the recognition they deserved at work, whereas 22.7% disagreed. Regarding salary adequacy, 52.3% were satisfied, while 47.7% were dissatisfied. When asked whether they would like to work in a paid job, 59.5% expressed willingness, while 40.5% did not want to work (Fig. 2).

### Reasons for not working

The most common reason for not working was health condition or disability (67.7%), followed by not being able to find a suitable job (20.7%) and insufficient transportation services (14.8%). Other notable reasons included lack of knowledge about how and where to seek employment (14.5%), not wanting to work (10.6%), and lack of access to potential workplaces (8.4%). Less frequently reported reasons included no financial need (6.1%), lack of assistive devices (5.8%), fear of losing disability benefits (3.6%), and personal or family-related restrictions, such as currently receiving education or vocational training (3.6%), family responsibilities (3.3%), or lack of permission from parents or spouse (1.9%) (Table 4).

**Table 4:** Reason for Not Working.

Reason for Not Working	Frequency (n)	Percentage (%)
Health condition or disability	242	67.7
Could not find a suitable job	74	20.7
Insufficient transportation services	53	14.8
Do not know how and where to look for a job	52	14.5
Do not want to work	38	10.6
Lack of access to potential workplaces	30	8.4
No financial need	22	6.1
Lack of assistive devices	21	5.8
Fear of losing disability benefits	13	3.6
Currently receiving education or vocational training	13	3.6
Personal family responsibilities	12	3.3
Parents or spouse did not allow me to work	7	1.9

## Discussion

In this study, we investigated the employment status, barriers to work, and associated factors among individuals with SCI. We found that only 12.9% of participants were engaged in paid employment, despite more than half (59.5%) expressing a willingness to work. This finding highlights a significant gap between the desire to work and actual employment rates in our population. Furthermore, education level, post-injury education, longer time since injury, and participation in VR were positively associated with employment, whereas SCI type and degree of injury were not. In this context, “SCI type” refers to paraplegia versus tetraplegia, and “degree” refers to complete versus incomplete injury. The lack of association with these neurological characteristics in our sample may suggest that, in this setting, environmental and systemic barriers (e.g., transport, workplace accessibility, and service availability) can outweigh lesion-related differences in shaping employment outcomes. The most frequently reported barrier to employment was health condition or disability (67.7%), followed by inability to find a suitable job (20.7%), transportation limitations (14.8%), and lack of knowledge about how and where to search for jobs (14.5%).

### Comparison with international and regional employment rates

Our observed employment rate is markedly lower than international benchmarks. In the multi-country InSCI Community Survey, the average employment rate was approximately 38%, although there was substantial variation across countries due to differences in social policies, labor-market structures, and accessibility infrastructure [7]. This discrepancy emphasizes the urgent need for country-specific strategies to promote workforce participation in our setting. Furthermore, according to the TURKSTAT, the national unemployment rate in July 2025 was approximately 8% (narrow definition) and 32% (broad definition) [8]. This indicates that the low employment rates observed in individuals with SCI may partly reflect the broader structural challenges of the Turkish labor market, where unemployment is already a significant societal issue.

A previous Turkish study reported an employment rate of 21%, which, while higher than ours, still reflects a substantial employment gap. That study identified female gender, higher education, post-injury education, younger age at injury, and longer time since injury as positive predictors of employment, whereas injury severity and level were not significant predictors [3]. These findings are mostly consistent with our results and underscore the pivotal role of educational attainment and adaptation over time in returning to work.

### Determinants of employment: education, time since injury, and vocational rehabilitation

Our results showed that years of education, particularly education obtained after SCI, were significantly higher among individuals who were employed. This aligns with established evidence that education is one of the most consistent and reproducible determinants of employment after SCI [9]. Anderson et al. also confirmed that higher education and greater functional independence significantly increase the likelihood of RTW [10].

Similarly, we observed that individuals who had lived longer with SCI were more likely to be employed. This may be explained by the gradual adjustment to disability, acquisition of new skills, and the development of strategies to overcome barriers over time, a trend reported in previous studies [3].

Participation in VR was significantly associated with current employment in our study. This supports the findings of Solheim et al. [11], who reported that early VR interventions and continuing in the same organization after injury significantly improve employment outcomes. Ottomanelli et al. demonstrated that community-based VR activities, particularly job development, placement, and follow-up support, were strongly associated with competitive employment, whereas traditional office-based counseling alone was insufficient to achieve similar outcomes [5]. Similarly, Roels et al. reported that while VR systems differ greatly between countries, common barriers such as lack of transportation, financial disincentives, and inadequate team education are universal challenges that must be addressed to optimize VR effectiveness [6]. These results suggest that expanding access to VR services and integrating them early in the rehabilitation process may reduce the employment gap in our population. Furthermore, tailoring VR strategies to address local systemic barriers—such as transport infrastructure—may enhance their impact, aligning with recommendations from international comparative studies.

Our cohort also included individuals with non-traumatic SCI (e.g., tumor, vascular, infection). For these etiologies, employment trajectories may be influenced not only by SCI-related disability but also by the underlying disease course and treatment burden (e.g., ongoing oncologic care, systemic comorbidity, fatigue), which could differentially affect work capacity and job continuity. Because the non-traumatic subgroup was relatively small, we were not able to conduct robust etiology-stratified analyses; future studies should examine traumatic and specific non-traumatic etiologies separately.

### Gender patterns

We found a significant gender difference, with women being more likely to be employed than men. This result contrasts with Solheim et al. [11], who reported no significant gender differences in Norway, and is inconsistent with Gündüz et al. [3], who reported that men have a higher employment rate. This pattern should be interpreted cautiously in light of the overall gendered employment structure in Turkey, where employment rates are substantially higher among men than women in national statistics. A plausible explanation is that the employed subgroup in our dataset was relatively small, and sampling/recruitment dynamics (e.g., differential survey participation among working individuals) may have influenced the observed distribution.

### Environmental and contextual barriers

Environmental barriers emerged as a prominent theme in our data. Among non-working participants, transportation difficulties (14.8%), lack of access to potential workplaces (8.4%), and limited knowledge of job search processes (14.5%) were frequently cited obstacles. These findings are highly consistent with Botticello et al. [12], who demonstrated that area-level socioeconomic status and environmental accessibility significantly influence employment prospects independent of individual health factors.

The InSCI framework also categorizes barriers into micro-level factors (e.g., assistive devices, transportation), meso-level factors (e.g., VR and healthcare services), and macro-level policies (e.g., labor-market regulations, disability benefits) [7]. Our findings align closely with this framework, particularly with regard to transport and access barriers, and they highlight the need for multi-level interventions. Notably, the distribution of barriers in our study spans all three levels of the InSCI framework, from individual health and assistive device issues to service-related barriers and broader policy-related challenges.

### Desire to work

Despite low employment rates, a substantial proportion of our participants (59.5%) reported a desire to work. This is consistent with other studies showing that many individuals with SCI wish to participate in the labor force, even when not currently employed [10].

Escorpizo et al. [13] demonstrated that employment is strongly associated with higher quality of life and better self-perceived health, with these effects being particularly pronounced in countries with higher gross domestic product.

Given that many of our participants face environmental barriers rather than a lack of motivation, enabling employment may have important downstream benefits for both quality of life and overall health.

Beyond obtaining employment, our work-environment indicators also speak to the quality and sustainability of work participation. A sizeable proportion of participants reported that workplace assistive-device needs were only partially met or not met, and nearly half indicated dissatisfaction with salary adequacy. Such findings may reflect gaps in reasonable accommodations and organizational support, which can affect not only hiring but also work retention, job stability, and inclusive participation over time. Strengthening workplace accommodation processes (assistive technology provision, accessibility, and supervisor/employer support) may therefore be as important as expanding job opportunities.

### Reasons for not working: convergence with other studies

The leading reason for not working in our study was health condition or disability, consistent with findings from numerous international studies [7, 10, 12].

However, a notable proportion of participants cited job unavailability, transportation barriers, and fear of losing disability benefits as reasons for not working. These factors closely mirror results from the InSCI Community Survey and a recent Romanian study, which also reported low VR participation (18.7%) and high expressed desire to return to work ( ≥ 60–80% depending on etiology) [14].

Although “fear of losing disability benefits” was selected by a small proportion of participants, this may underestimate benefit-related concerns. This item was captured via a fixed-choice list and may be sensitive to social desirability or to participants’ awareness of benefit rules. Qualitative work and more detailed benefit-related questioning may better characterize how financial disincentives influence return-to-work decisions after SCI.

Our findings, supported by the existing literature, have several important implications for both clinical practice and policy development. First, expanding opportunities for post-injury education and skills training is essential to enhance the employability of individuals with SCI. Individuals with higher education levels, particularly those who attained additional education after injury, had significantly higher employment rates, indicating that supporting individuals to pursue further education can play an important role in improving workforce participation. Second, strengthening VR services is critical. Given that participation in VR was significantly associated with employment, scaling up individualized programs that include career counseling, job-search skills training, and active employer engagement may help bridge the gap between willingness to work and actual employment. Additionally, addressing transportation and workplace accessibility barriers should be a policy priority. Many participants in our study identified these factors as major obstacles, aligning with previous international findings that environmental barriers profoundly shape employment outcomes. Improving public transport systems, enhancing workplace accessibility, and ensuring availability of assistive devices could reduce these constraints. Furthermore, policy reforms are needed to mitigate the fear of losing disability benefits, which was cited by some participants as a reason for not seeking employment. A more flexible benefits system that allows for gradual workforce reintegration without penalizing individuals financially may encourage labor-force participation. Finally, gender-sensitive strategies should be developed to address the unique barriers faced by women with SCI. The significant gender disparity observed in our study suggests that interventions must be tailored to the cultural and societal context to promote equitable employment opportunities.

By implementing these strategies, it may be possible to reduce the substantial employment gap, improve the quality of life of individuals with SCI, and create a more inclusive and productive workforce.

This study has limitations. Participants were recruited predominantly through Istanbul-based centers and a national association, which may limit generalizability to the broader Turkish SCI population despite attempts to include individuals from different regions. Employment was assessed via self-report and largely binary categorization (currently in paid employment vs not), which may not capture important job-quality dimensions such as full- versus part-time work, formal versus informal employment, job security, or working hours. In addition, the employed subgroup was relatively small (46/357), which may reduce statistical power and yield less stable comparisons for some categorical variables with sparse cells. Finally, the inclusion of non-traumatic etiologies introduces heterogeneity in clinical course; etiology-specific analyses were not feasible in the present dataset and warrant future investigation.

## Conclusion

Our study contributes valuable insights into the complex interplay of individual, environmental, and systemic factors associated with employment among people with SCI. While the majority of participants expressed a desire to work, structural and environmental barriers prevented workforce participation. Addressing these barriers through educational initiatives, VR expansion, accessibility improvements, and supportive policies has the potential to significantly improve employment outcomes for individuals with SCI.

## Data Availability

The datasets generated and/or analyzed during the current study are available from the corresponding author upon reasonable request. The data are not publicly available due to ethical restrictions and the sensitive nature of participant-level survey data.
